# Supplementary material for: Is there a moderate range of impact of financialization on corporate R&D?
Source: PLoS One. 2021 Jun 30;16(6):e0253380. doi: 10.1371/journal.pone.0253380 (PMC8244881; doi:10.1371/journal.pone.0253380)
Supplement: Attachment — Submitted filename: Response to Reviewers.docx [file pone.0253380.s002.docx]

**Reply to the reviewers’ comments for the manuscript “Is there a moderate range of impact of financialization on corporate R&D?”**

Dear Editors and Reviewers:

Thank you very much for your letter and comments concerning our manuscript entitled“Is there a moderate range of impact of financialization on corporate R&D?”. The comments are valuable and very helpful for us to revise and improve our paper, as well as the important guiding significance to our researches. We have studied comments carefully and have made correction which we hope meet with approval. Revised portion are marked in red in the paper. The main corrections in the paper and the responds to the reviewers’ comments are as following:

**Reviewer #1**: Paper seems well drafted and researched.

Though authors are does not seem native English speakers, language seems good.

I have a few concerns:

1、What was the basis of selection of specific financial variables? Was there is possibility of many other financial variables? What is rational?

**Reply:** Thanks for your concerns and comments. According to Accounting Standards for Business Enterprises, enterprise assets can be divided into operational assets and financial assets. Financial assets are mainly used for investment and financing activities of enterprises. Therefore, when measuring corporate financialization, financial assets items and quasi-financial assets items in the balance sheet of enterprises should be selected. The selection of financial variables in the control variables is mainly based on the relationship between variables and R&D. Although there are many factors influencing R&D, we choose financial variables that have a significant impact on R&D according to relevant references [38-40].

References:

38.	He JJ, Tian X. The dark side of analyst coverage: The case of innovation. J Financ Econ. 2013;109(3):856-78. doi: <https://doi.org/10.1016/j.jfineco.2013.04.001>.

39.	Hong M, Drakeford B, Zhang K. The impact of mandatory CSR disclosure on green innovation: evidence from China. Green Finance. 2020;2(3):302-22. doi: <https://doi.org/10.3934/GF.2020017>.

40.	Su K, Liu H. Financialization of manufacturing companies and corporate innovation: Lessons from an emerging economy. Manag Decis Econ. 2021. doi: <https://doi.org/10.1002/mde.3278>

2、What is use of this research to Industry and academia?

**Reply：**Thanks for your comments. To Industry, this research provides a reference for enterprises to allocate financial assets rationally, and provides an appropriate range of the proportion of corporate financial assets held by enterprises, which promotes R&D, and achieves sustainable development goals. To academia，the current research mainly focuses on the promotion or inhibition effect of financialization on corporate R&D, this research provides a theoretical basis for the dynamic relationship between financialization and R&D, and provides empirical evidence for the moderate range of the impact of financialization on R&D.

3、What are future directions of research?

**Reply:** Thanks for your comments. We have limitations in the measurement of financialization, and the relationship between financialization and R&D needs to be further studied. Future research can optimize the measurement of financialization and study the moderate range of the impact of corporate financialization on R&D under the influence of macroeconomic environment. For specific explanation, the research prospect is added at the end of this paper (Line 526-529, Line 533-535). The content of the modification has been marked in red in the article.

4、Verify calculations for correctness and verify panel threshold regression model of Hansen and restated model. Do all calculations and remove errors if any.

**Reply:** Thanks for your concerns and comments. To ensure the accuracy of the results in this article, we rechecked the original data, the calculated data, the STATA procedures we used and reworked the results. After checking, there is no calculation error in this paper, and the result of panel threshold regression model of Hansen is correct.

5、I will advise you improve literature or background study. Refer a few articles to improve, https://doi.org/10.21314/JOP.2020.245 and http://dx.doi.org/10.1504/IJMFA.2018.095976

**Reply:** Thanks for your advice. In order to fully explain the significance of our research, we add the negative impact of financial development, the importance of corporate R&D, and the financing constraints faced by corporate R&D (line 47-52, line 73-77). We also added references (reference 3, 9, 10, 11, 12, 15, 16, 17).The content of the modification has been marked in red in the article.

**Reviewer #2:** The paper investigates a widely researched topic from a new aspect, how the financialization impacts on corporate R&D. The literature is well processed and analyzed. A (general) part of the literature review is included in the Introduction (without formulationg a subchapter), the other part is to find in the theoratical foundation (second chapter). The sources are analysed well, in a critical and comprehensive way.

The authors selected the appropriate statistical methodology toolset (panel threshold regression model) and applied well. The results are logical, supported by the methodology and datasets. The conclusions are based on the results, reflecting to the question set in the paper's title.

As a summary, in my opinion it is an excellent academic paper.

1、My only suggestion is to include the limitations of the research at the end of the paper.

**Reply:** Thanks for your comments. We have limitations in the measurement of financialization, and the relationship between financialization and R&D needs to be further studied. For specific explanation, the research limitations are added at the end of this paper (Line 523-526, Line 529-533). The content of the modification has been marked in red in the article.
